# *Trichinella spiralis* excretory/secretory proteins mediated larval invasion via inducing gut epithelial apoptosis and barrier disruption

**DOI:** 10.1371/journal.pntd.0012842

**Published:** 2025-01-23

**Authors:** Qi Qi Lu, Wen Wen Zheng, Zhao Yu Zhang, Pei Kun Cong, Xin Guo, Yao Zhang, Xin Zhuo Zhang, Shao Rong Long, Ruo Dan Liu, Zhong Quan Wang, Jing Cui

**Affiliations:** Department of Parasitology, School of Basic Medical Sciences, Zhengzhou University, Zhengzhou, China; NIAID-ICER, INDIA

## Abstract

**Background:**

Intestinal larva invasion is a crucial step of *Trichinella spiralis* infection. Intestinal infective larvae (IIL) and their excretory/secretory proteins (ESP) interact with gut epithelium, which often results in gut epithelium barrier injuries. Previous studies showed when *T*. *spiralis* invaded intestinal epithelium cells, the IIL ESP disrupted the tight junctions (TJs) of Caco-2 monolayer, but the mechanism is not clear. The IIL ESP might cause gut epithelial apoptosis, weaken the gut barrier and aid the larval invasion. The aim of this study was to investigate whether *T*. *spiralis* IIL ESP participate in enterocyte apoptosis and disrupt gut epithelial barrier to promote the larval invasion.

**Methodology/Principal findings:**

Cell viability was assessed by CCK-8 assay and the results showed that 200 μg/ml of IIL ESP incubated with Caco-2 cells for 18 h inhibited the Caco-2 cell viability. The results of trans-epithelial electrical resistance (TEER) and FITC-dextran showed that IIL ESP decreased the TEER, increased FITC-dextran flux in Caco-2 monolayer. qPCR, Western blot and immunofluorescence test (IFT) showed that IIL ESP decreased the mRNA and protein expression of TJs (ZO-1, E-cad, Occludin and Claudin-1). The IIL ESP-induced Caco-2 cell apoptosis was observed by DAPI, Hoechst 33358, TUNEL and Annexin V/PI staining. Besides, flow cytometry revealed an increasing apoptosis rate in Caco-2 cells after the IIL ESP treatment. qPCR and Western blot analysis indicated that IIL ESP activated caspases (Caspase 3, Caspase 9 and Caspase 8), up-regulated the pro-apoptotic factors (Bax and Cytochrome c) and down-regulated the anti-apoptosis molecule Bcl-2. Interestingly, pretreatment of Caco-2 cells with apoptosis inhibitor Z-VAD-FMK abrogated and recovered the barrier function of Caco-2 monolayer destroyed by IIL ESP. Furthermore, the Z-VAD-FMK pretreatment also impeded the *in vitro* larva invasion of Caco-2 monolayer.

**Conclusions:**

*T*. *spiralis* IIL ESP induced gut epithelial apoptosis, reduced the TJs expression, damaged gut epithelial integrity and barrier function, and promoted larval invasion. These findings provided a basis of further understanding the interaction mechanism between *T*. *spiralis* and host gut epithelium, and they were valuable to the development new prevention and therapeutic strategy of early *T*. *spiralis* infection.

## Author summary

*Trichinella spiralis* is a nematode of genus *Trichinella* that infects a variety of mammals worldwide, and intestinal larva invasion is a crucial step of *T*. *spiralis* infection. *T*. *spiralis* intestinal infective larvae (IIL) and their excretory/secretory proteins (ESP) interact with the host gut epithelium, which promoted the larval invasion, but the IIL invasive mechanism is not clear. Apoptosis is involved in multitudinous pathological process in parasite infection. In this study, the effect of *T*. *spiralis* IIL ESP on Caco-2 monolayer barrier function was assessed by using trans-epithelial electrical resistance (TEER), FITC-dextran, qPCR, Western blot and IFT. The results showed that IIL ESP decreased the TEER, increased FITC-dextran flux in Caco-2 monolayer, decreased the expression of tight junctions (TJs; ZO-1, E-cad, Occludin and Claudin-1). These results suggested that IIL ESP damaged the integrity of intestinal epithelium barrier. The IIL ESP-induced Caco-2 cellular apoptosis was observed by DAPI, Hoechst 33358, TUNEL and Annexin V/PI staining. Besides, a flow cytometry exhibited an increasing apoptosis rate of Caco-2 cells treated with IIL ESP. qPCR and Western blot results showed that IIL ESP activated caspases (Caspase 3, Caspase 9 and Caspase 8), up-regulated the pro-apoptotic factors (Bax and Cytochrome c), down-regulated the anti-apoptosis molecule Bcl-2. Pretreatment with apoptosis inhibitor Z-VAD-FMK abrogated and recovered the barrier function of Caco-2 monolayer destroyed by IIL ESP. The inhibitor pretreatment also impeded the *in vitro* larva invasion of Caco-2 monolayer. These findings demonstrated that IIL ESP mediated larva invasion via inducing gut epithelial apoptosis and damaging gut barrier integrity.

## Introduction

*Trichinella spiralis* is a nematode of genus *Trichinella* that infects a variety of mammals worldwide [1]. There are about 10, 000 recorded cases of human *Trichinella* infection per year in the world. The European Food Safety Authority and the European Centre for Disease Prevention reported 398 confirmed cases of human trichinellosis in 27 countries of Europe Union during the period of 2018–2022 [2]. Total of 6662 human trichinellosis cases in Argentina during 2012–2018 and 258 cases of human trichinellosis during 2005–2015 in Chile were confirmed [3]. Eight human trichinellosis outbreaks with 479 cases and 2 deaths were reported in China during the period of 2009–2020 [4].

*T*. *spiralis* infection was due to eating raw or uncooked meat containing infective muscle larvae (ML). The infected meat was digested in host stomach, and the ML was released from their collagen capsule and activated into intestinal infective larvae (IIL) by bile or intestinal contents in gut. The IIL invades intestinal epithelium and developed to adult worms (AW) [5, 6]. After being mated, the pregnant female adults produce the newborn larvae (NBL). The NBL invade into host’s capillary vessels and are transported to the skeletal muscles, then encapsulated to complete the lifecycle [7]. During the lifecycle, the IIL are first contacted and interacted with intestinal epithelium cells (IEC), the intestinal epithelium is the first barrier for defending against *T*. *spiralis* invasion and infection [8,9]. However, the mechanism of the interaction between *T*. *spiralis* and gut epithelium is not completely elucidated [10, 11].

The IIL are the first *T*. *spiralis* invasive phase to invade the host’s enteral mucosa in the lifecycle, and their excretory/secretory proteins (ESP) contain a variety of functional proteins [12]. Our previous studies revealed that when *T*. *spiralis* IIL invaded and migrated in the IEC monolayer, the IIL ESP also remained in the IEC cytoplasm [10, 13]. Furthermore, we found that *T*. *spiralis* infection and IIL ESP disrupted the intestinal tight junctions (TJs) barrier integrity and increased the permeability of Caco-2 monolayer and gut mucosa [14]. Nevertheless, the mechanism of the destructive effect of IIL ESP to the gut barrier integrity is not clear. The IIL ESP might cause gut epithelial apoptosis, weaken the gut barrier and aid the larval invasion.

Genetically programmed cell death (apoptosis) is not only a basic physiological process which participates in the maintenance of homeostasis and proper development of an organism, but also involved in multitudinous pathological process containing parasites infection [15]. Cell apoptosis has two main pathways: intrinsic pathway and extrinsic pathway. Intrinsic apoptosis occurs through internal pathways and is triggered by any stimuli that cause oxidative stress, mitochondrial dysfunction and DNA damage. Mitochondrial damage leads to mitochondrial outer membrane permeabilization, and promotes the release of cytochrome c into the cytoplasm. The released cytochrome c then binds to APAF-1 which recruits pro-Caspase 9 to form a complex called the "apoptosome", and then triggers the apoptotic cascade. Extrinsic pathway is induced by ligand binding to death receptors which include tumor necrosis factor receptor 1 (TNFR1), Fas and TRAIL receptor. Caspase 8 and Caspase 10 initiate death receptor-mediated extrinsic apoptosis; whereas Caspase 9 initiates the withdrawal of dependence receptor ligand-mediated extrinsic apoptosis.

Intestinal epithelial integrity plays an important role for defensing the intestinal parasite invasion. It is known that numerous pathogens (such as *Entamoeba* and *Giardia*) impair the epithelial barrier function by regulating apoptosis mechanism [16,17]. *Giardia* broke the epithelial barrier function via activating caspase 3-mediated intestinal cell apoptosis [18, 19]. *Entamoeba*-induced apoptosis of host enterocyte promoted the parasites infection to gut mucosa [20]. *Blastocyst*-induced apoptosis of human IEC disrupted epithelial barrier and ZO-1 organization in a caspase 3- and 9-dependent pattern [21]. It has been reported that *T*. *spiralis* infection at intestinal stage induced the apoptosis in the villous lamina propria cells [22]. *T*. *spiralis* infection could induce the murine IEC apoptosis by activating the endoplasmic reticulum stress (ERS) [23]. Recent studies showed that exosomes derived from *T*. *spiralis* ML also induced apoptosis and disrupted the intestinal barrier [24]. However, it is not clear whether *T*. *spiralis* IIL ESP disrupt intestinal epithelial barrier and promote larval invasion of intestinal epithelium through inducing enterocyte apoptosis. There are no reports on the relation between the *T*. *spiralis-*related enterocyte apoptosis and gut epithelium barrier integrity in the literatures.

The aim of the present study was to investigate whether *T*. *spiralis* IIL ESP induce the host’s enterocyte apoptosis and disrupt gut epithelial barrier to mediate the larval invasion of gut epithelium.

## Materials and methods

### Ethics statement

This study was performed according to the National Guidelines for Experimental Animal Welfare (Minister of Science and Technology, People’s Republic of China, 2006). All animal experiments in this study were approved by the Life Science Ethics Committee of Zhengzhou University (No. ZZUIRB GZR 2021–0044).

### Parasites and animals

The parasite used in this research was *Trichinella spiralis* strain (ISS534), which was collected from a naturally infected swine in Henan province, China and passaged in BALB/c mice in our laboratory [25]. The BALB/c mice were provided by the Experimental Animal Center of Zhengzhou University, Henan province, China.

### Cell culture

Human colon cancer cell line Caco-2 cells were purchased from Cell Bank of Shanghai Institute of Biological Sciences, Chinese Academy of Sciences. Caco-2 cells were cultured in Dulbecco’s modified eagle medium (DMEM; Servicebio, China) containing 10% fetal bovine serum (Biological Industries, Israel), 100 μg/ml streptomycin, 100 U/ml penicillin and 100 μM non-essential amino acids (Solarbio, China) at 37°C under 5% CO_2_ [26, 27].

### Preparation of *T*. *spiralis* IIL ES proteins

The IIL worms were obtained from the intestines of mice orally infected with 5000 *T*. *spiralis* ML at 6 hours post infection (hpi) according the method mentioned previously. The IIL excretory/secretory proteins (ESP) were prepared as described previously [28, 29]. After washing with sterile PBS and RPMI-1640 medium with 100 μg/ml streptomycin and 100 U/ml penicillin, the IIL at 5000/ml were cultured in the medium at 37°C under 5% CO_2_ for 18 h. The culture supernatant was collected and concentrated by Amicon Ultra centrifugal filtration device (MW cut-off value: 3 kD) at 4°C [12, 30]. The IIL ESP concentration was measured by Bradford method and stored at -80°C.

### Cell counting Kit-8 (CCK-8) assay

To determine the IIL ESP effect on the Caco-2 cell ability, CCK-8 assay was performed [31]. Caco-2 cells (3 × 10^4^ cells/well) were seeded on a 96-well plate, and then incubated with different concentrations of IIL ESP (0, 25, 50, 100 and 200 μg/ml) for various time (0, 3, 6, 12, 18, 24 and 48 h). And then, 10 μl/well of CCK-8 solution (TargetMol, USA) was added to the medium and incubated for another 1 h at 37°C with 5% CO_2_. The absorbance at 450 nm was measured with a plate reader (Tecan, Switzerland).

### Trans-epithelial electrical resistance (TEER) assay

In a previous study, we found that incubation of Caco-2 monolayer with IIL ESP for 2 h damaged the monolayer integrity and reduced the TJs expression [10]. But, whether apoptosis induced by IIL ESP affected the epithelail barrier function is not clear. The TEER was usually used to evaluate the integrity of intestinal epithelium barrier [19]. Caco-2 cells were seeded and cultured to the cell confluence in a trans-well insert (6.5 mm diameter, 0.4 μm pore size, BIOFIL, China), and the TEER of Caco-2 monolayer was monitored by a Millicell-ERS volt-ohmmeter (Millipore, USA). Resistance value = (measured value—blank value) × film bottom area. When the TEER was stability (above 300 Ω·cm^2^), various concentration of IIL ESP was added to the apical side of Caco-2 monolayer and incubated at 37°C for 18 h, and the TEER was measured again. TEER value was normalized to its initial value before treatment and expressed as TEER (%) [32, 33].

### Determination of paracellular permeability

Paracellular permeability of Caco-2 monolayer was assessed by using FITC-dextran diffusion assay [14]. Caco-2 cells were seeded and cultured to the cell confluence in a trans-well insert. Caco-2 monolayer was first incubated with 120 μM Z-VAD-FMK (apoptotic inhibitor; APE×BIO, USA) at 37°C for 1 h, followed by the incubation with IIL ESP at 37°C for 18 h, then 0.5 mg/ml of FITC-dextran (MW: 4 kDa, FD4, Sigma, USA) dissolved in medium were added into the apical side of the monolayer [18]. Meanwhile, the medium without FD4 was added to the basal chamber and incubated at 37°C for 2 h [33, 34]. The medium in the basal chamber was collected and the absorbance value at 485–520 nm was measured using a microplate reader (Molecular Devices, USA) [11].

### Real-time quantitative PCR (qPCR) assay

The qPCR assay was performed to ascertain the transcription level of apoptosis and TJs proteins in Caco-2 monolayer as reported previously [6,35]. Caco-2 monolayer was incubated with 200 μg/ml of IIL ESP for 18 h. The total RNA of Caco-2 cells was extracted by TRIzol reagent (Sangon Biotech, China), its quality and concentration were measured using NanoDrop 2000 (Thermo Fisher, USA) and then reverse-transcribed into cDNA according to the instructions of PrimeScriptRT reagent Kit (TaKaRa, Japan). qPCR assay was carried out using Applied Biosystems 7500 Fast System (Life Technologies, USA) [31]. Specific primers for the TJs and apoptosis-related genes examined in this study were synthesized by Sangon Biotech. The primers sequences of the related genes investigated in this study were listed in Table 1. Relative mRNA expression of these genes in this study were normalized to GAPDH as an internal control and calculated according to the 2^−ΔΔCt^ method [12, 36].

**Table 1:** Primer sequences of human gut tight junctions and apoptosis-related genes for qPCR.

Gene names	Primer	Sequence (5′-3′)	GenBank no.
ZO-1	Forward	CGGTCCTCTGAGCCTGTAAG	NM_001330239.4
	Reverse	GGATCTACATGCGACGACAA	
E-cad	Forward	GCCTCCTGAAAAGAGAGTGGAAG	NM_131820.1
	Reverse	TGGCAGTGTCTCTCCAAATCCG	
Occludin	Forward	ATGGCAAAGTGAATGACAAGCGG	XM_026274194.1
	Reverse	CTGTAACGAGGCTGCCTGAAGT	
Claudin-1	Forward	GTCTTTGACTCCTTGCTGAATCTG	NM_021101.5
	Reverse	CACCTCATCGTCTTCCAAGCAC	
GAPDH	Forward	TGTGTCCGTCGTGGATCTGA	NM_002046.7
	Reverse	TTGCTGTTGAAGTCGCAGGAG	
Caspase 3	Forward	GAAATTGTGGAATTGATGCGTGA	NM_001354777.2
	Reverse	CTACAACGATCCCCTCTGAAAAA	
Caspase 8	Forward	TTTCTGCCTACAGGGTCATGC	NM_001080124.2
	Reverse	TGTCCAACTTTCCTTCTCCCA	
Caspase 9	Forward	GTTTGAGGACCTTCGACCAGCT	NM_001229.5
	Reverse	CAACGTACCAGGAGCCACTCTT	
PARP	Forward	CGGAGTCTTCGGATAAGCTCT	NM_001618.4
	Reverse	TTTCCATCAAACATGGGCGAC	
Cytochrome c	Forward	ATGAAGTGTTCCCAGTGCCA	NM_018947.6
	Reverse	CTCTCCCCAGATGATGCCTTTG	
Bax	Forward	TCAGGATGCGTCCACCAAGAAG	NM_001291428.2
	Reverse	TGTGTCCACGGCGGCAATCATC	
Bcl-2	Forward	ATCGCCCTGTGGATGACTGAGT	NM_000633.3
	Reverse	GCCAGGAGAAATCAAACAGAGGC	

### Immunofluorescence test (IFT)

The IFT was performed to investigate the expressions of Caco-2 monolayer TJs proteins as described previously [37, 38]. In brief, Caco-2 cells grown on glass coverslip were washed thrice with PBS and fixed by 4% paraformaldehyde for 20 min at room temperature [39]. After permeabilizing with 0.25% Triton X-100 for 10 min, the cells were blocked with 5% bovine serum albumin (BSA) in PBS at 37°C for 1 h. Then the cells were probed at 4°C overnight with the primary antibodies against ZO-1(1:500, Servicebio), E-cad (1:500, Abcam), Occludin (1:100, Invitrogen) and Claudin-1 (1:20, Invitrogen). After washing with PBS thrice, the cell monolayers were incubated with CY-3-labeled anti-rabbit IgG antibody for 1 h at 37°C, followed by a DAPI staining for the nuclei. Visualization of cells was performed with fluorescence microscopy (Olympus, Japan) [11, 14].

### Western blot analysis

To assess the expression level of apoptosis and TJs proteins of Caco-2 cells treated with IIL ESP, Western blot was performed as described before [31, 40]. In brief, the total cellular soluble proteins were prepared by using RIPA lysis buffer containing 1% protease inhibitor, separated by SDS-PAGE and then transferred onto polyvinylidene fluoride (PVDF) membranes (Millipore, USA) [41]. After incubation with 5% skim milk dissolved in TBS containing 0.05% Tween-20 (TBST) at room temperature for 1 h, the membranes were cut into the strips, and then incubated for 16 h at 4°C with the primary rabbit or mouse anti-human antibodies against Caspase 3 and cl-Caspase 3 (1:500, Abmart, China), Caspase 8 and cl-Caspase 8 (1:5000, Proteintech, China), Caspase 9 and cl-Caspase 9 (1:5000, Proteintech, China), PARP and cl-PARP (1:500, Abmart, China), Bax (1:1000, Abmart, China), Bcl-2 (1:500, Abmart, China), Cytochrome c (1:1000, Abmart, China), ZO-1 (1:1000, Servercebio, China), E-cad (1:20000, Abcam, UK), Occludin (1:125, Invitrogen, USA), Claudin-1 (1:125, Invitrogen, USA), and Tubulin (1:5000, Abmart, China). Furthermore, to further confirmed that IIL ESP induces Caco-2 cell apoptosis other than the alternative cell death pathways (such as pyroptosis), the expression level of pyroptosis protein Gasdermin D (GSDMD) in IIL ESP-treated Caco-2 cells was also ascertained on Western blot, the primary rabbit anti-human antibodies against pyroptosis protein GSDMD-FL and GSDMD-N (1:1000, Abmart, China) were used. The strips were washed with TBST buffer and then incubated with secondary antibodies (goat anti-rabbit or anti-mouse IgG conjugated with HRP) at room temperature for 1 h. After washing with TBST, the strips were colored using enhanced chemiluminescence (ECL) reagent (Meilunbio, China), and detected via a chemiluminescence image system (Tanon, China) [39, 42]. The relative intensity of protein bands were analyzed using the Image J software (National Institutes of Health, USA) and normalized by using tubulin band [43, 44].

### Measurement of cell apoptosis

DAPI (Solarbio, China) staining was performed to confirm the cellular apoptosis by observing the morphology of cell nucleus, and Hoechst 33258 (Beyotime, China) staining was carried out to observe the intensity of blue fluorescence [24, 45]. Additionally, TUNEL staining was conducted to ascertain the apoptotic cells using One-step TUNEL Apoptosis Assay Kit (Abbkine, China) as the manufacturer’s protocol [46]. Briefly, Caco-2 cells were fixed with 4% paraformaldehyde and then stained with Terminal dUTP nick-end labeling (TUNEL) detection solution at 37°C for 2 h in dark. Following this, the cell nuclei were stained with DAPI and observed with a fluorescence microscope (Olympus, Japan). Furthermore, apoptotic cells were also stained using AbFluor 488-Annexin V and propidium iodide (PI) via an Annexin V-AbFluor 488/PI kit (Abbkine, China); then observed with a fluorescence microscope (Olympus) and analyzed by a flow cytometry (FACScan; BD Biosciences, San Jose, CA) [47]. The apoptosis rate was calculated by adding up early and late apoptosis rate.

### The *in vitro* larval invasion test

To ascertain the effect of apoptosis inhibitor on *T*. *spiralis* invasion of intestinal epithelium, an *in vitro* invasion assay was performed as described earlier [48, 49]. *T*. *spiralis* muscle larvae (ML) were collected from the skeletal muscle of infected mice by artificial digestion with 1% pepsin (1:3000) and 1% hydrochloric acid, and they were activated to the IIL by 5% mouse bile at 37°C for 2 h. Caco-2 cell monolayer were pretreated with 120 μM apoptosis inhibitor Z-VAD-FMK at 37°C for 1 h, and then treated with IIL ESP for 18 h at 37°C. Finally, 100 IIL suspended in 1.75% agarose mixed with RPMI-1640 medium were added onto the Caco-2 monolayer and incubated for 2 h at 37°C. The larval invaded the monolayer was observed and counted under an optical microscope (Olympus, Japan) [50, 51].

### Statistical analysis

All data in this research are presented as means ± standard deviation (SD) and analyzed with SPSS 21.0 software. Data were analyzed by two tailed Student t-test or one-way ANOVA. Statistical significance was considered when *P* < 0.05.

## Results

### *T*. *spiralis* IIL ESP inhibited the Caco-2 cell viability

**Fig 1:**
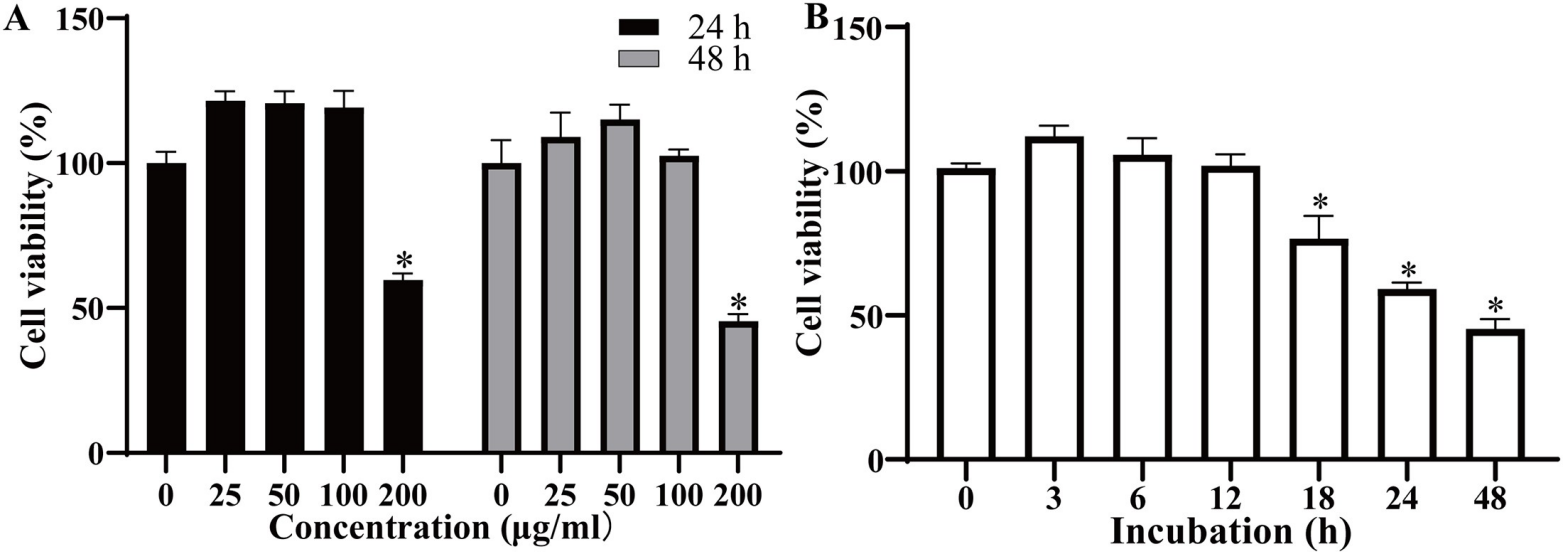
*T*. *spiralis* IIL ESP effect on Caco-2 cell viability. **A:** 0, 25, 50, 100 and 200 μg/ml of IIL ESP were incubated with Caco-2 cells for 24 h and 48 h. **B:** Caco-2 cells were incubated with 200 μg/ml of IIL ESP for different time. The data are presented as mean ± standard deviation (SD) of three independent tests. *P < 0.05 compared to the blank control (0 μg/ml or 0 h) group.

In order to evaluate the effect of IIL ESP on the cell viability of Caco-2, CCK-8 assay was carried out. When different concentrations of IIL ESP were incubated with Caco-2 cells, the cell viability was significantly inhibited by 200 μg/ml of IIL ESP at 24 h and 48 h (*F*_24 h_ = 173.1, *F*_48 h_ = 114.2, *P* < 0.0001) (Fig 1A). When Caco-2 cells were incubated with 200 μg/ml IIL ESP for 0–48 h, there was a significant inhibitory of cell viability at 18, 24 and 48 h (*F* = 97.81, *P* < 0.0001) (Fig 1B). These findings showed that *T*. *sprialis* IIL ESP inhibited the cell viability after 18 h incubation in an incubation time-dependent manner. Therefore, in the following experiments, 200 μg/ml of IIL ESP were chosen as the co-incubating concentration with Caco-2 cells and incubation time was 18 h.

### *T*. *spiralis* IIL ESP disrupted the integrity and barrier function of Caco-2 monolayer

**Fig 2:**
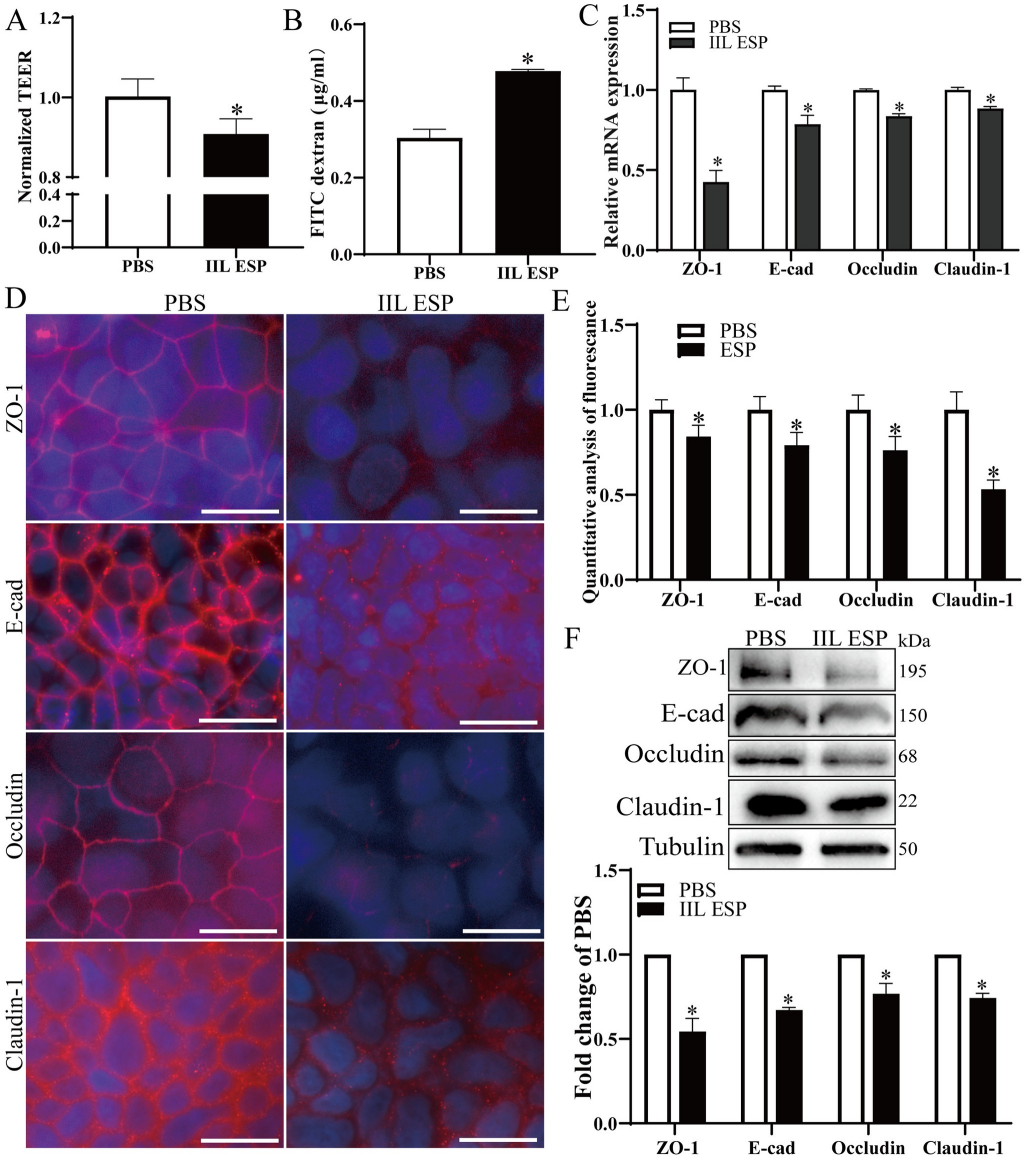
*T*. *spiralis* IIL ESP reduced TJ protein expression and destroyed the integrity of Caco-2 monolayer. **A:** IIL ESP decreased the TEER of Caco-2 monolayer. **B:** IIL ESP increased FITC-dextran (4 kDa) flux through the monolayer. **C:** qPCR analysis of the mRNA expression level of TJs in IIL ESP-treated Caco-2 monolayer. **D:** IFT analysis of the TJs protein expression in IIL ESP-treated Caco-2 monolayer, scale bar: 20 μm. **E:** Quantitative analysis of the TJs fluorescence intensity. **F:** Western blot analysis of the TJs protein expression in IIL ESP-treated Caco-2 cells. Data are presented as mean ± SD of three independent assays. *P < 0.05 compared to PBS group.

To investigate the effect of IIL ESP on gut epithelail barrier function, Caco-2 cells were treated with 200 μg/ml of IIL ESP for 18 h at 37°C, and the TEER was measured to evaluate the cell monolayer integrity. As shown in Fig 2A, IIL ESP decreased TEER by 9.41% (*t* = 2.802, *P* < 0.05). Meanwhile, the permeability of FITC-dextran in Caco-2 monolayer was also significantly increased after IIL ESP treatment compared to the PBS group (*t* = 13.43, *P* < 0.0001) (Fig 2B). qPCR analytical results showed that the mRNA expression levels of four TJs genes (ZO-1, E-cad, Occludin and Claudin-1) in IIL ESP treatment group were decreased by 57.57% (*t* = 9.690, *P* < 0.01), 21.37% (*t* = 6.087, *P* < 0.01), 16.30% (*t* = 17.55, *P* < 0.0001) and 11.60% (*t* = 13.431, *P* < 0.01), respectively, compared to the PBS group (Fig 2C). The expression and location of four TJ proteins in IIL ESP-treated monolayer were also observed by a fluorescence microscope. The results revealed that the fluorescence intensity of ZO-1, E-cad, Occludin and Claudin-1 was reduced by 15.74% (*t* = 3.047, *P* < 0.05), 20.86% (*t* = 3.321, *P* < 0.05), 23.84% (*t* = 3.482, *P* < 0.05) and 46.72% (*t* = 6.817, *P* < 0.01) respectively after treatment with IIL ESP (Fig 2D and 2E). Moreover, Western blot showed that protein expression levels of ZO-1, E-cad, Occludin and Claudin-1 were decreased by 0.599 (*t* = 14.437, *P* < 0.01), 0.494 (*t* = 14.061, *P* < 0.01), 0.393 (*t* = 8.295, *P* < 0.05) and 0.358 (*t* = 3.421, *P* < 0.05) folds separately following IIL ESP treatment (Fig 2F). Overall, these findings indicated that IIL ESP reduced the protein expression of intestinal epithelium TJs, damaged the epithelial barrier integrity and increased the permeability.

### *T*. *spiralis* IIL ESP induced Caco-2 cell apoptosis

**Fig 3:**
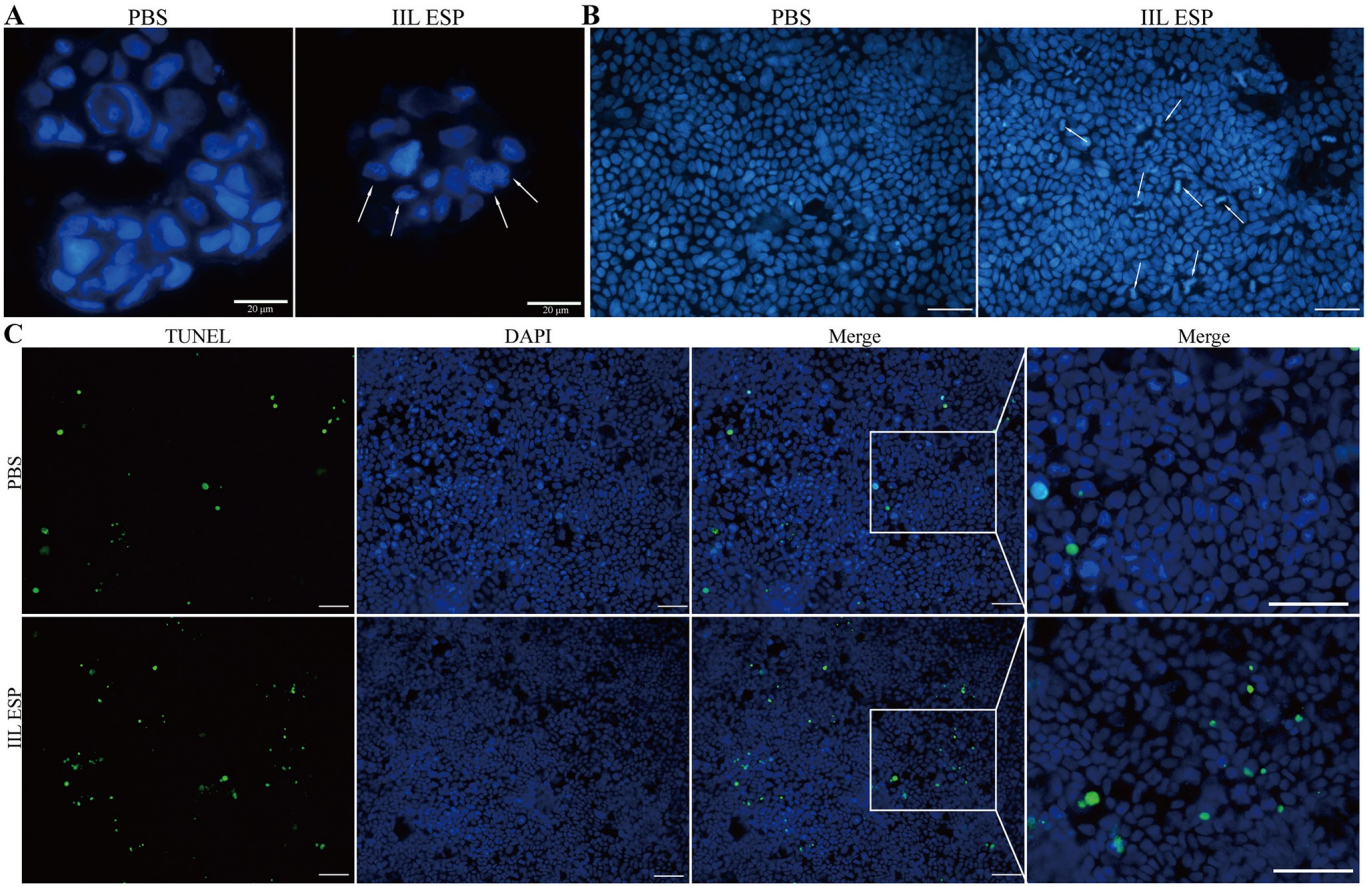
*T*. *spiralis* IIL ESP induced Caco-2 cell apoptosis stained by DAPI, Hoechst 33258 and TUNEL staining. Caco-2 cells were incubated with 200 μg/ml IIL ESP for 18 h. **A:** The nuclear morphology stained by DAPI (blue), white arrows indicating the broken nuclei, scale bar: 20 μm; **B:** Hoechst 33258 staining, the apoptotic cells exhibited deep blue staining and nuclear condensation (white arrows), scale bar: 50 μm; **C:** TUNEL staining of Caco-2 cells, the apoptotic cells were stained green, scale bar: 50 μm.

**Fig 4:**
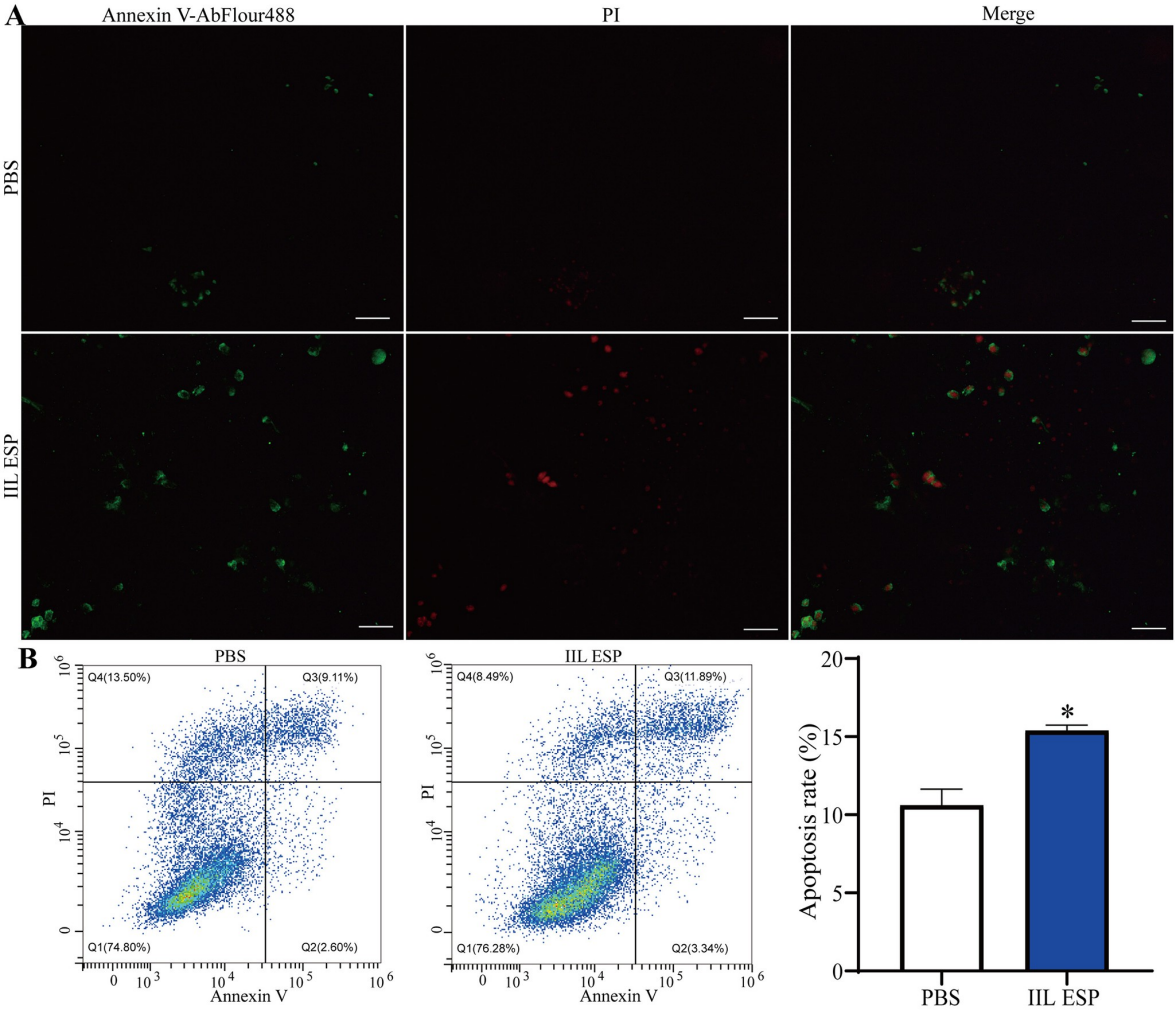
*T*. *spiralis* IIL ESP-induced apoptosis stained by Annexin V-AbFlour488/PI staining. Annexin V-AbFlour488 and PI staining was performed to distinguish the apoptosis cells. Annexin V-AbFlour488 positive cells were apoptosis cells and PI positive cells were death cells, while the positive cells of both Annexin V-AbFlour488 and PI were late apoptosis cells. In addition, Annexin V-AbFlour488 and PI negative cells were normal cells. **A:** Annexin V-AbFlour488 (green) and PI (red) staining for apoptosis detection via a fluorescence microscope. **B:** Annexin V and PI staining for apoptosis detection via flow cytometry, and the percentage of Annexin V-AbFlour488 positive cells was shown in right bar graph. Scale bar: 50 μm. Data are presented as mean ± SD of three independent tests. *P < 0.05 compared to the PBS group.

After Caco-2 cells were incubated with 200 μg/ml IIL ESP for 18 h and stained with DAPI, Hoechst 33258, TUNEL and Annexin V/PI, the cells were observed by a fluorescence microscope. The results showed that the cells of PBS group exhibited a relative intact morphology, but the cells in IIL ESP treatment group showed nuclear fragmentation, nuclear pyknosis, and chromatin border sets (Fig 3A). Moreover, the normal live cell nuclei stained by Hoechst 33258 were homogeneous blue, while the IIL ESP-treated cells had more apoptotic cells with deep staining and nuclear condensation (Fig 3B). TUNEL staining revealed the apoptosis of Caco-2 cells treated with the IIL ESP. Compared to the PBS group, the IIL ESP treatment group exhibited an increased number of apoptotic cells (Fig 3C). Furthermore, the apoptotic cells were stained with Annexin V and the cell apoptosis rate was analyzed by flow cytometry. The apoptotic cells in the IIL ESP group were stained green by Annexin V as shown in Fig 4A. After staining with Annexin V and PI, Caco-2 cells were detected by flow cytometry. The cell apoptosis rate of the PBS group was 10.62%, and the cell apoptosis rate of the IIL ESP group was 15.40%, which is 0.45 folds higher than the PBS group (*t* = 7.797, *P* < 0.01) (Fig 4B). Taken together, these results indicated that *T*. *spiralis* IIL ESP induced the apoptosis of Caco-2 cells.

### *T*. *spiralis* IIL ESP activated apoptosis signaling pathway in Caco-2 cells

**Fig 5:**
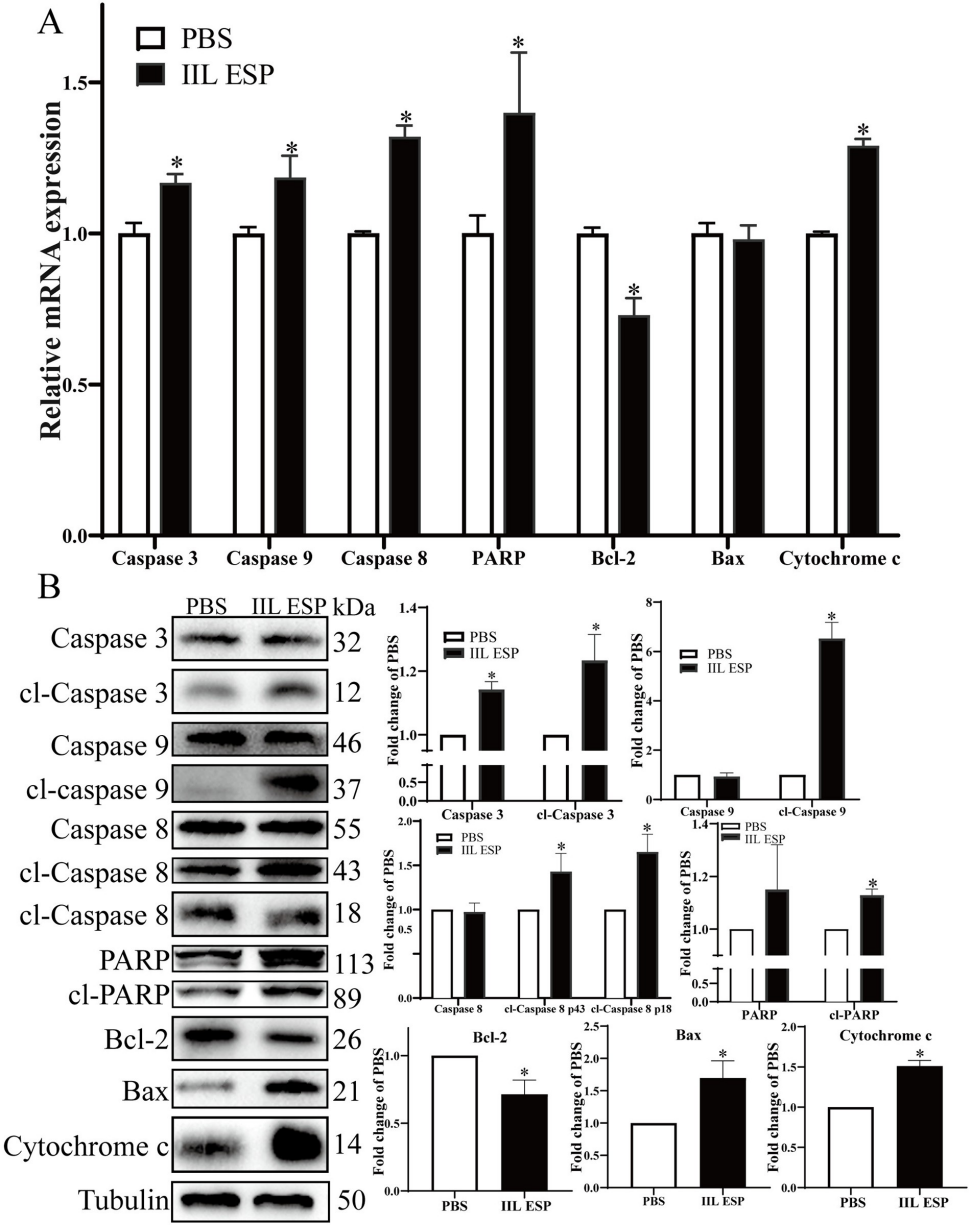
Changes of mRNA and protein expression levels of apoptotic genes of Caco-2 cells treated with *T*. *spiralis* IIL ESP. **A:** qPCR analysis of the mRNA expression levels of apoptotic genes of IIL ESP-treated Caco-2 cells. **B:** Western blot analysis of apoptotic proteins expression levels of IIL ESP-treated Caco-2 cells. Data are presented as mean ± SD of three independent experiments, *P < 0.05 compared to the PBS group.

To ascertain the mechanism of IIL ESP-induced the cell apoptosis, qPCR and western blot assays were performed to detect the expression levels of apoptosis pathway molecules. Caco-2 monolayer was treated with 200 μg/ml of IIL ESP at 37°C, 5% CO_2_ for 18 h, and then the mRNA and proteins were isolated, and analyzed by qPCR and Western blot. Compared to the PBS group, the IIL ESP significantly increased the mRNA expression of pro-apoptotic genes of Caspase 3, Caspase 9, Caspase 8, PARP and Cytochrome c respectively by 0.167 (*t* = 6.434, *P* < 0.01), 0.185 (*t* = 4.334, *P <* 0.05), 0.320 (*t* = 14.70, *P* < 0.0001), 0.398 (*t* = 3.315, *P* < 0.05) and 0.290 (*t* = 21.755, *P* < 0.0001) folds in Caco-2 cells, but the IIL ESP decreased the mRNA expression level of anti-apoptotic gene Bcl-2 by 0.270 (*t* = 7.857, *P <* 0.01). Nevertheless, the mRNA expression level of pro-apoptotic gene Bax has no significant change compared to the PBS group (*t* = 0.610, *P* > 0.05) (Fig 5A).

Western blot analysis showed that the Caco-2 cells treated with the IIL ESP presented significantly increased protein expression levels of Caspase 3, cl-Caspase 3 p12, cl-caspase 9, cl-caspase 8 p43 and p18, cl-PARP, Bax and Cytochrome c by 0.142 (*t* = 9.782, *P* < 0.05), 0.234 (*t* = 4.967, *P* < 0.05), 5.522 (*t* = 14.557, *P* < 0.0001), 0.429 (*t* = 3.587, *P* < 0.05) and 0.652 (*t* = 5.692, *P* < 0.05), 0.128 (*t* = 9.549, *P* < 0.01), 0.698 (*t* = 4.556, *P* < 0.05) and 0.510 (*t* = 9.609, *P* < 0.01) folds, respectively; whereas the IIL ESP significantly decreased the expression levels of the anti-apoptotic protein Bcl-2 by 0.285 fold (*t* = 4.728, *P* < 0.05) (Fig 5B). These findings indicated that IIL ESP activated the apoptosis-related pathway and induced Caco-2 cell apoptosis.

Additionally, to investigate whether the IIL ESP induce pyroptosis of Caco-2 cells, the expression level of cell pyroptosis executor GSDMD in IIL ESP treated Caco-2 cells was assessed on Western blot. The results revealed that expression level of both the full length GSDMD (GSDMD-FL) and cleaved GSDMD (GSDMD-N) had no significant change after IIL ESP treatment compared to the PBS group (S1 Fig) (*t*_GSDMD-FL_ = 0.127, *P* > 0.05; *t*_GSDMD-N_ = 0.053, *P* > 0.05). These results further verified that IIL ESP induced Caco-2 cell apoptosis rather than alternative cell death pathways (pyroptosis).

### Apoptotic inhibitor abrogated and reduced the IIL ESP-damaged TJs and barrier junction of Caco-2 monolayer

**Fig 6:**
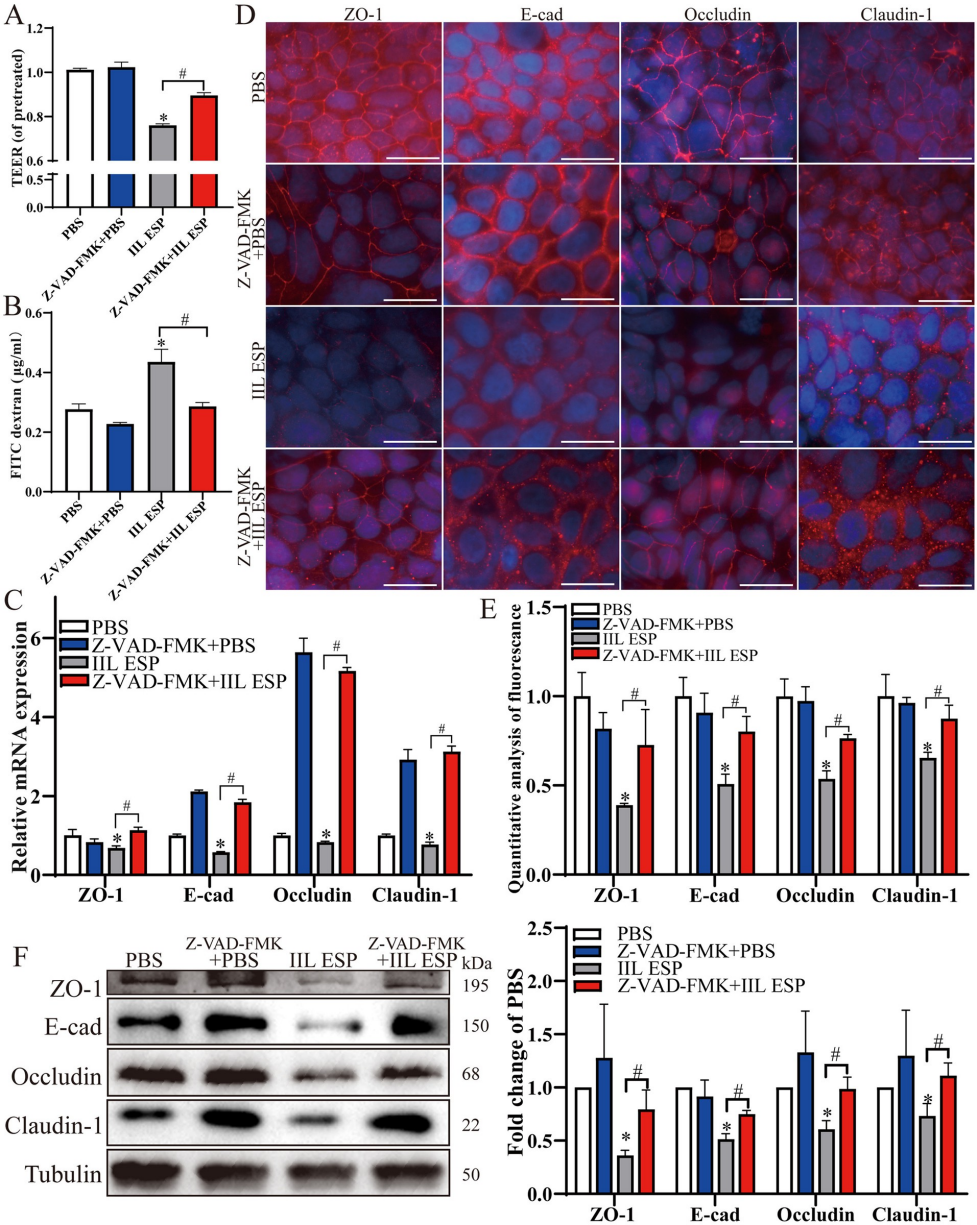
Apoptosis inhibitor prevented the decrease of IIL ESP-induced TJs expression and alleviated barrier disruption of Caco-2 monolayer. **A:** Apoptosis inhibitor Z-VAD-FMK pretreatment increased the IIL ESP-decreased TEER. **B:** Z-VAD-FMK pretreatment reduced the IIL ESP-increased FITC-dextran flux. **C:** qPCR analysis of the Z-VAD-FMK pretreatment increasing mRNA expression level of TJs in IIL ESP-treated Caco-2 monolayer. **D and E:** fluorescence microscopy of Z-VAD-FMK pretreatment recovering the expression of TJs proteins in IIL ESP-treated Caco-2 monolayer (scale bars: 20 μm) and quantitative analysis of the TJs fluorescence intensity. **F:** Western blot analysis of TJs expression in Z-VAD-FMK pretreated- Caco-2 monolayer after incubation with IIL ESP. Data are presented as mean ± SD of three independent experiments. *P < 0.05 compared to PBS group, ^#^*P* < 0.05 compared to the individual IIL ESP group.

To further study the role of IIL ESP-induced apoptosis on the disruption of epithelial cell barrier, Caco-2 monolayer was first pretreated by an apoptotic pharmacological inhibitor Z-VAD-FMK, following by re-incubated with the IIL ESP. We found a 13.44% increase of TEER (*t* = 16.295, *P* < 0.0001) and a significant decrease of FITC-dextran flux (*t* = 5.855, *P* < 0.01) in Z-VAD-FMK-pretreated IIL ESP group, compared to the alone IIL ESP group (Fig 6A and 6B). As shown in Fig 6C, Z-VAD-FMK pretreatment abrogated and increased the IIL ESP-reduced mRNA expression levels of TJs genes ZO-1 (*t* = 8.662, *P* < 0.01), E-cad (*t* = 27.939, *P* < 0.0001), Occludin (*t* = 83.716, *P* < 0.0001) and Claudin-1 (*t* = 26.686, *P* < 0.0001). Also, Z-VAD-FMK pretreatment rescued and increased the IIL ESP-suppressed TJs protein expression of ZO-1, E-cad, Occludin and Claudin-1 by 0.871 (*t* = 2.945, *P* < 0.05), 0.579 (*t* = 5.002, *P* < 0.01), 0.424 (*t* = 8.038, *P* < 0.01) and 0.335 (*t* = 4.701, *P* < 0.01) folds respectively, which was displayed in IFT analysis (Fig 6D and 6E). In addition, Western blot results exhibited a distinct increased expression levels of ZO-1, E-cad, Occludin and Claudin-1 by 1.200 (*t* = 3.955, *P* < 0.05), 0.545 (*t* = 6.325, *P* < 0.01), 0.624 (*t* = 4.778, *P* < 0.01) and 0.520 (*t* = 3.950, *P* < 0.05) folds in inhibitor pretreated-Caco-2 cells after incubation with IIL ESP relative to the alone IIL ESP group (Fig 6F). These results further confirmed that IIL ESP induced-apoptosis resulted in the disruption of Caco-2 monolayer barrier integrity.

### Apoptosis inhibitor impeded *T*. *spiralis* larvae invading to Caco-2 monolayer

**Fig 7:**
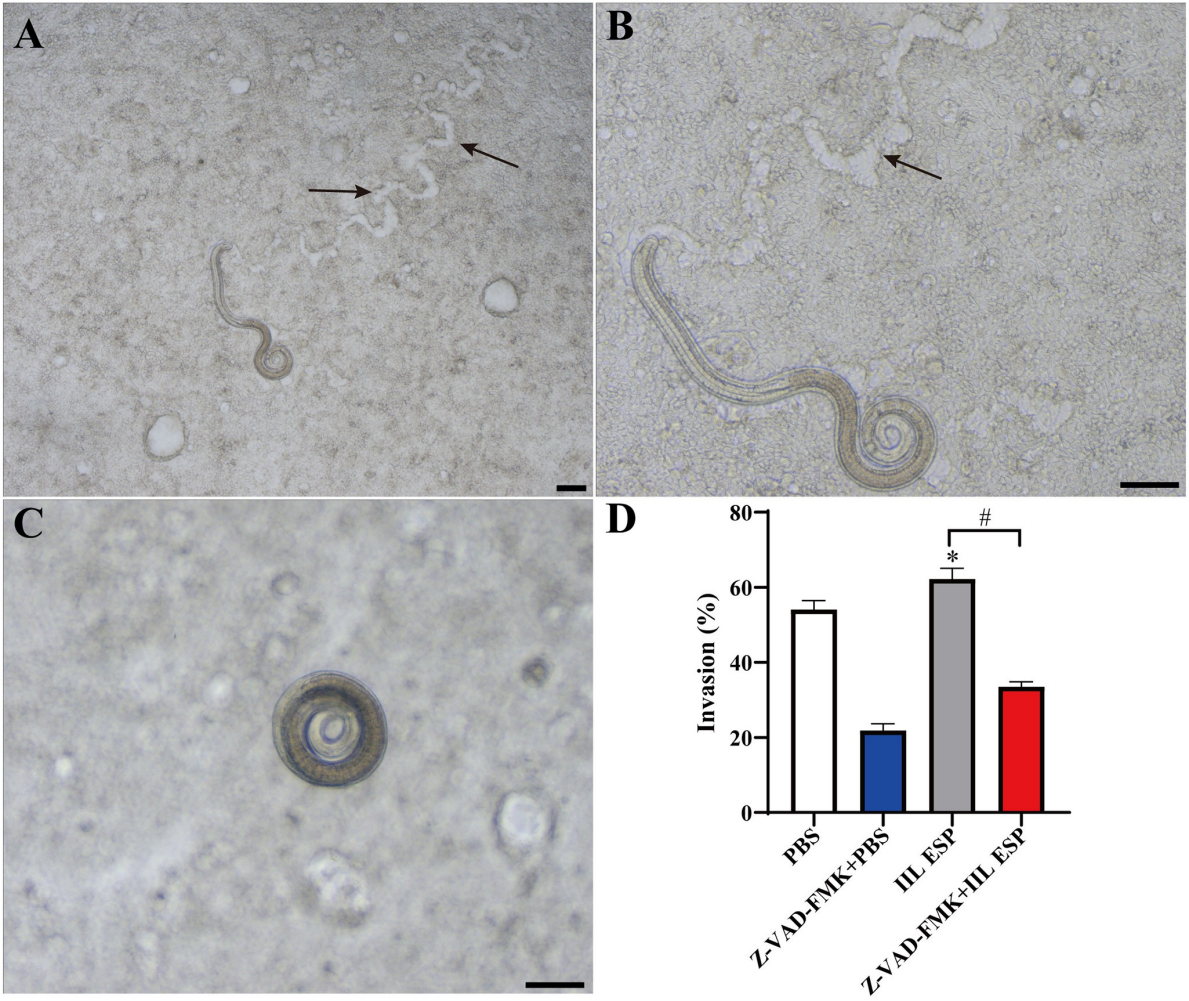
Apoptosis inhibitor impeded larval invasion of Caco-2 monolayer. Caco-2 monolayers were pretreated with Z-VAD-FMK and then incubated with *T*. *spiralis* IIL ESP for 18 h, one hundred IIL were added and incubated at 37°C for another 2 h. The larval invasion was observed under a microscope. **A and B:** larva invaded Caco-2 monolayer, and the larval migratory traces were indicated by arrows. **C:** non-invaded larva exhibited a spiral coil. **D:** IIL ESP facilitated the larval invasion while apoptosis inhibitor Z-VAD-FMK pretreatment suppressed the larval invasion. Data are presented as mean ± SD of three independent assays. Scale bars: 100 μm. *P < 0.05 compared to PBS group, ^#^*P* < 0.05 compared to the only IIL ESP group.

The *in vitro* larval invasion assay was performed to investigate the effect of IIL ESP-induced apoptosis on *T*. *sprialis* larval invading gut epithelium. Caco-2 cells were first pretreated with apoptosis inhibitor Z-VAD-FMK, then treated with the IIL ESP for 18 h and incubated with 100 IIL for 2 h. The invasion or non-invasion larvae were captured and counted under a microscope. The larva invaded into Caco-2 monolayer was as shown in Fig 7A and 7B. The arrows were indicated the worm migratory traces. However, the non-invaded larva appeared the spiral coil as shown in Fig 7C. As shown in Fig 7D, the IIL ESP significantly promoted the larval invasion by 8.18% (*t* = 3.831, *P* < 0.05) compared to the PBS group. While Caco-2 cells were pretreated with apoptosis inhibitor Z-VAD-FMK and incubated with the IIL ESP, the larval invasion shown an obvious decrease of 28.69% (*t* = 16.066, *P* < 0.0001), compared to the only IIL ESP group. The results suggested that IIL ESP promoted the larval invasion, whereas inhibition of gut epithelial apoptosis clearly impeded the larval invasion of gut epithelium.

## Discussion

The IIL is the first and key worm stage invading host intestinal epithelium in *T*. *spiralis* infection, the IIL ESP are directly exposed to the host intestinal mucosa and interacted with gut epithelium [10,12]. The IIL ESP is continuously secreted at the intestinal stage of *T*. *spiralis* infection, but the detailed amount of IIL ESP produced within an infected host is unclear. Therefore, a high concentration (200 μg/ml) of *T*. *spiralis* IIL ESP was used to investigate its *in vitro* interaction with Caco-2 cell monolayer in the present study. The basic intestinal barrier is established and maintained by enterocyte and TJs proteins [52]. Our previous studies have found that *T*. *spiralis* IIL ESP disrupted the gut epithelium barrier and increased the permeability of Caco-2 monolayer [10]. However, the molecular mechanism of *T*. *spiralis* IIL ESP affecting the permeability and TJs of gut epithelial barrier is unclear. In the present study, we evaluated the permeability and TJs protein expression in IIL ESP-treated Caco-2 monolayer. The results of TEER and FITC-dextran showed that 200 μg/ml of IIL ESP (a protein dose inhibiting the cell viability) increased the Caco-2 monolayer permeability. Moreover, both mRNA and protein expression levels of TJs proteins (ZO-1, E-cad, Occludin and Claudin-1) were obviously reduced in IIL ESP-treated Caco-2 cells. These finding demonstrated that *T*. *spiralis* IIL ESP damaged gut epithelium integrity and barrier function when the cell viability was inhibited [23].

The gut epithelial barrier is the first line to defense against the entrance of antigens, toxic and harmful substances in intestinal tract, serves as not only a physical barrier but also a participant in intestinal mucosal immunity [53]. Thus, the balance between cell survival and cytotoxicity, apoptosis, injury and inflammatory responses is critical for maintaining normal gut epithelial barrier function [52]. It had been reported that *T*. *spiralis* infection and its ESP induce the IEC apoptosis [22,47]. Our previous studies demonstrated that *T*. *spiralis* ESP remained in the cells when the larvae invaded the IEC [10,13]. In this work, the results of CCK-8 kit assay shown that *T*. *spiralis* IIL ESP decreased the Caco-2 cell viability. DAPI and Hoechst 33258 staining found that the Caco-2 cell nuclei were damaged and displayed an apoptotic state after the IIL ESP treatment. An increasing ratio of apoptotic cells in the IIL ESP-treated Caco-2 were also detected by TUNEL staining. Flow cytometry results showed a significant increase of apoptotic rate of the IIL ESP-treated Caco-2 cells. These findings demonstrated that the IIL ESP resulted in the IEC apoptosis.

It has been reported that *T*. *spiralis* infection induced the ERS in the mouse jejunum and the *T*. *spiralis* Kazal-type serine protease inhibitor (TsKaSPI) induced ERS in the porcine small intestinal epithelial cells (IPECs) [47]. Other studies found that *T*. *spiralis* ML ESP increased the expression of pro-apoptotic proteins and down-regulated anti-apoptotic proteins [54,55]. In programmed cell death, the caspases family of cysteine proteases is a key regulator. A cascade of caspase activation mediates cellular destruction and signal transduction during apoptosis [56,57]. Cell apoptosis is divided into two kinds: intrinsic apoptosis and extrinsic apoptosis. Intrinsic apoptosis can be induced by DNA damage, metabolic stress, hypoxia and other factors. Intrinsic apoptosis begins with the change of mitochondrial outer membrane permeability (MOMP) caused by Bax and Bak which are the pro-apoptotic members of BCL2 family, and leads to the release of mitochondrial proteins such as Cytochrome c into the cytosol. Cytosolic cytochrome c interacts with APAF1, which recruits pro-Caspase 9 to form the apoptosome. Caspase 3, Caspase 6 and Caspase 7 are considered the common effector caspases for both extrinsic and intrinsic apoptosis. Additionally, the extrinsic pathway can trigger intrinsic mitochondrial apoptosis through the generation of truncated BID (tBID) by activating Caspase 8. tBID can further translocate to mitochondria and cause MOMP through the activation of Bax and Bak. Extrinsic apoptosis is induced by the addition of death receptor ligands or by the withdrawal of dependence receptor ligands. Caspase 8 and Caspase 10 initiate death receptor-mediated extrinsic apoptosis; whereas Caspase 9 initiates the withdrawal of dependence receptor ligand-mediated extrinsic apoptosis. Pro-Caspase 8 and pro-Caspase 10 are enzymatically inactive until they interact with Fas-associated via death domain (FADD), which is activated upon binding to cell death receptors responding to their ligands [58,59]. In intrinsic apoptosis, the Bcl-2 protein family is the pivotal regulators. While Caspase 8 plays an important role in extrinsic pathway [57]. To further assess the relationship between *T*. *spiralis* IIL ESP and apoptosis and explore which apoptosis pathways were involved, the expressions of apoptosis-related genes and proteins were examined by qPCR and Western blot in the present study. The qPCR results showed a significant increase of pro-apoptotic genes (Caspase 3, Caspase 9, Caspase 8, PARP, Bax and Cytochrome c), and a significant decrease of anti-apoptotic genes such as Bcl-2 at mRNA level in IIL ESP-treated Caco-2 cells. The Bcl-2 protein family is regarded as primary intracellular regulator for apoptotic process and is related with the mitochondria-dependent apoptosis pathway. Cell survival controlled by apoptotic process is decided by the balance between the up-regulation and down-regulation of pro-apoptotic and anti-apoptotic proteins [60]. Furthermore, activation of caspase is necessary for apoptosis. The increased protein expression levels of cleaved-Caspase 3, cleaved-Caspase 9 and cleaved-Caspase 8 in IIL ESP-treated Caco-2 cells indicated that Caspase 3, Caspase 9 and Caspase 8 were activated by the IIL ESP. Meanwhile, PARP as a downstream substrate of Caspase 3 was cleaved, further suggesting the activation of Caspase 3. When the serum-free medium was used to the mock treated control group (e.g., the PBS group) and Caco-2 cells were serum-starved, some caspases (such as caspase 8 and 3) were cleaved, and the cleaved products (cl-caspase) were detected in the PBS group [61,62].

Additionally, the pro-apoptotic proteins (Bax and Cytochrome c) were significantly up-regulated by the IIL ESP. Furthermore, the increasing expression of Bax, Cytochrome c and cl-Caspase-9 and the decreasing expression of Bcl-2 induced by the IIL ESP suggested that the intrinsic pathway was activated in Caco-2 cells. While, the activating of Caspase 8 indicated that the extrinsic pathway also been activated by the IIL ESP. The results suggested that *T*. *spiralis* IIL ESP activated both intrinsic and extrinsic pathways and resulted in apoptosis of gut epithelial cells [63]. Nevertheless, which protein molecules in IIL ESP take part in the apoptosis is not undefined in this study, and it needs to be identified and characterized in future experiment.

Apoptosis is involved in many intestinal infectious diseases and induces intestinal epithelium injury [15]. Some research found that deoxynivalenol produced by a fungus disrupted intestinal epithelial barrier by inducing IEC apoptosis. *Blastocystis* disrupted the gut epithelium barrier and ZO-1 in a caspase 3- and 9-dependent mode, and the apoptosis inhibitor recovered the barrier function of Caco-2 monolayer [21]. *Giardia lamblia* infection induced the enteral epithelial apoptosis and increased the gut epithelial permeability which was suppressed by Caspase 3 inhibitor [17, 18, 64]. However, the effect of *T*. *spiralis* IIL ESP on apoptosis of enteral epithelium cells and the relationship among cell apoptosis, epithelium barrier and *T*. *spiralis* invasion has not been elucidated up till now. In this research, we found that apoptosis inhibitor Z-VAD-FMK reduced gut epithelium permeability, recovered and strengthened barrier function of Caco-2 monolayer. Moreover, the transcription expression levels of the TJs proteins were regained and increased in the Z-VAD-FMK pretreated-Caco-2 monolayer following the IIL ESP treatment relative to alone IIL ESP group. Furthermore, apoptosis inhibitor Z-VAD-FMK pretreatment also suppressed *T*. *spiralis* larval invasion of Caco-2 monolayer *in vitro*. The results implied that gut epithelium apoptosis inhibition attenuated intestinal epithelial barrier damage caused by IIL ESP and impeded the larval invasion of gut epithelium, demonstrated that *T*. *spiralis* IIL ESP mediated larval invasion through inducing enteral epithelium apoptosis and disrupting enteral epithelial barrier integrality [19]. However, there are still some limitations in this study. Although our results confirmed that *T*. *spiralis* IIL ESP induced the gut epithelial cell apoptosis to disrupt gut barrier integrity, but which protein molecules in IIL ESP participate in the apoptosis are not undefined. It needs to be identified and characterized in future experiment. In addition, the protective roles of apoptosis inhibitor on *T*. *spiralis*-infected mice should be investigated in future study.

In conclusion, our results illustrated the important role of cell apoptosis induced by *T*. *spiralis* IIL ESP in disrupting intestinal epithelial barrier integrity and larval invasion. It is the first indication that *T*. *spiralis* IIL ESP reduced the TJs expression, damaged gut barrier function and promoted larval invasion via inducing gut epithelial apoptosis. These findings provided a basis of further understanding the interaction mechanism between *T*. *spiralis* and host gut epithelia, and they were valuable to the development new preventive and therapeutic strategy of early *T*. *spiralis* infection.

## Supporting information

S1 Fig Western blotting identification of expression level of pyroptosis protein Gasdermin D (GSDMD) in IIL ESP-treated Caco-2 cells. The expression level of both the full length GSDMD (GSDMD-FL) and cleaved GSDMD (GSDMD-N) in Caco-2 cells after IIL ESP treatment had no significant change compared to the PBS group. Data are presented as mean ± SD of three independent experiments.
(TIF)

S1 Table The raw data for building the graphs in this study. (XLSX)
