# Supplementary figures and images for: Trichinella spiralis excretory/secretory proteins mediated larval invasion via inducing gut epithelial apoptosis and barrier disruption
Source: PLoS Negl Trop Dis. 2025 Jan 23;19(1):e0012842. doi: 10.1371/journal.pntd.0012842 (PMC11793818; doi:10.1371/journal.pntd.0012842)

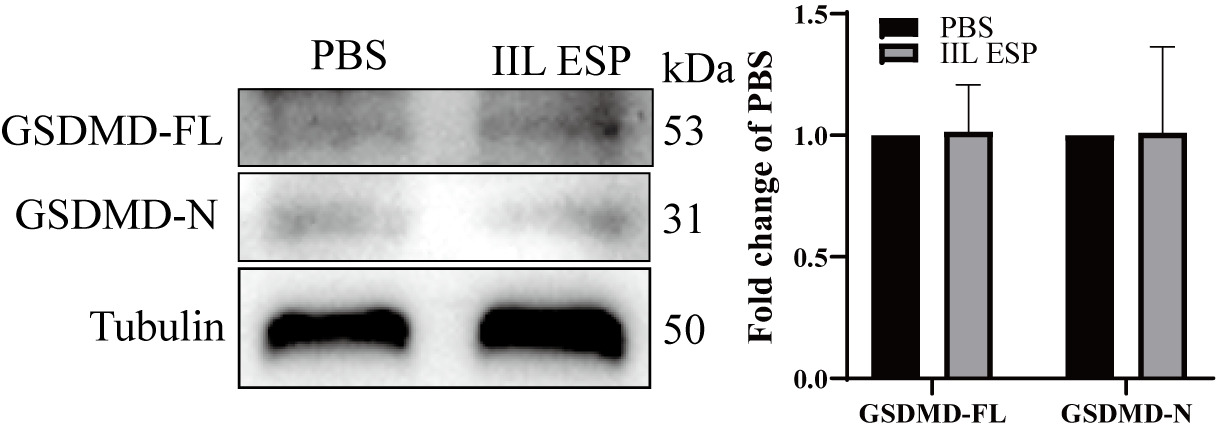

Supplement: S1 Fig — The expression level of both the full length GSDMD (GSDMD-FL) and cleaved GSDMD (GSDMD-N) in Caco-2 cells after IIL ESP treatment had no significant change compared to the PBS group. Data are presented as mean ± SD of three independent experiments. (TIF) [file pntd.0012842.s001.tif]
